# Clinical features and prognostic factors of cryptococcal infections in HIV-infected patients: a 10-year study from an infectious disease specialist hospital

**DOI:** 10.3389/fcimb.2024.1407807

**Published:** 2024-08-14

**Authors:** Fang-Fang Dai, Jin-Li Lou, Yan-Hua Yu, Ming Chen, Xin-Xin Lu

**Affiliations:** ^1^ Beijing Tongren Hospital, Capital Medical University, Beijing, China; ^2^ Department of Clinical Laboratory, Beijing Youan Hospital, Capital Medical University, Beijing, China

**Keywords:** human immunodeficiency virus, *Cryptococcus*, acquired immune deficiency syndrome, prognosis, antiretroviral therapy

## Abstract

**Background:**

Cryptococcosis is an invasive infection that commonly affects immunosuppressed individuals, especially patients with HIV infection. Cryptococcal infection in HIV-infected patients should be considered a major health concern because it is associated with high morbidity and mortality rates. In this study, we aimed to evaluate the clinical characteristics and prognostic factors of cryptococcal infections in human immunodeficiency virus (HIV)-infected patients to facilitate effective clinical management and improve patient outcomes.

**Methods:**

We reviewed and analyzed the clinical data and relevant laboratory test results of HIV-infected patients with positive cryptococcal cultures and reserved strains between 2013 and 2023 from Beijing Youan Hospital affiliated to Capital Medical University. The clinical characteristics and laboratory test results of the patients were compared, and the correlation between parameters and the prognoses of the patients at different observation timepoints (3, 6, 9, and 12 months) was analyzed.

**Results:**

A total of 76 patients (70 males and six females; median age, 37 years) were included in this study. The results indicated that the later the initiation of antiretroviral therapy (ART) after the diagnosis of HIV infection (> 6 months), the higher the probability of death. Analysis of the correlation between the time of ART initiation and the timing of treatment for cryptococcal infections showed that the time of ART initiation was strongly related to survival at different timepoints. Initiation of ART time within 0-4 weeks, 4-6 weeks and more than 6weeks of starting treatment for *Cryptococcus* infection was associated with a lower mortality rate at 12-month, the 3-month, 6- and 9-month follow-up timepoint separately.

**Conclusions:**

Although cryptococcal infection in HIV-infected patients continues to be a challenging and intricate issue, ART is a key factor that affects its prognosis. The later ART is started, the worse the prognosis of the infection. The time of ART initiation and the timing of treatment for cryptococcal infections should be further refined and balanced based on different clinical courses. Thus, clinicians should pay closer attention to cryptococcal infections in patients with HIV infection and initiate ART based on the patient’s clinical condition.

## 1 Introduction

*Cryptococcus* belongs to the phylum Basidiomycetes, which is classified under the kingdom Fungi. *Cryptococcus* is widely distributed in nature and has a spherical or oval cell wall surrounded by a capsule. *Cryptococcus* includes more than 30 species; however, two main species complexes cause diseases in humans: *Cryptococcus neoformans* and *Cryptococcus gattii* (Ferry et al., 2015; May et al., 2016; Soare et al., 2019). *Cryptococcus neoformans* is the primary etiological agent of cryptococcal disease and a leading cause of AIDS-related infection (World Health Organization, 2018; Rajasingham et al., 2022). *Cryptococcus* is usually transmitted through the inhalation of fungal spores and can colonize the human respiratory tract. The inhaled Cryptococcus spores or yeast cells may be cleared by the host or enter a latent phase. Once the immune system is weakened, *Cryptococcus* may activate, proliferate, and spread through the blood to other organs in the body. The interaction between *Cryptococcus* and the host’s immune response is complicated. In addition, the mechanism of recognition of *Cryptococcus* by the immune system and the activation and effects of the immune response on the fungus are not completely clear.

Due to its neurophilic nature, *Cryptococcus* is more likely to break through the blood–brain barrier than other pathogens (Silva et al., 2020; Chen et al., 2022; Hamed et al., 2023), eventually leading to cryptococcal meningitis, which can be fatal if untreated. Despite the increasing efforts to expand access to antiretroviral therapy (ART) in recent years, the incidence of cryptococcal meningitis in HIV-infected patients has not significantly declined (Hevey et al., 2019; Dantas et al., 2023). Cryptococcal infection in HIV-infected patients should be viewed as a major health concern because of its clinically challenging nature, widespread incidence, and high morbidity and mortality rates. Extensive research and focused education regarding its management are essential for improved patient outcomes (Ndlovu et al., 2020; Sun et al., 2020; Chang et al., 2024). Therefore, we conducted the present study to investigate the clinical features and prognostic factors of cryptococcal infections in HIV-infected patients.

## 2 Materials and methods

### 2.1 Study population and data collection

We reviewed the clinical data of HIV-infected patients with cryptococcal infection confirmed through cerebrospinal fluid (CSF) or blood culture between 1 March 2013 and 1 March 2023 at Beijing Youan Hospital of Capital Medical University, China. All the included patients had accessible medical records and their *Cryptococcus* isolates were reserved. Patients with inaccessible medical records or without *Cryptococcus* isolates were excluded from the study. The clinical data of the patients were extracted from their electronic medical records. The data collected included demographic characteristics, medical history, and laboratory test results. The patients were categorized into “survival” or “death” groups based on their survival status at the 3, 6, 9, and 12-month follow-up timepoints.

### 2.2 Identification of *Cryptococcus* species

*Cryptococcus* species were identified using MALDI-TOF mass spectrometry on a Bruker Microflex instrument (Bruker Daltonics GmbH, Bremen, Germany) according to the manufacturer’s recommendations (Tarumoto et al., 2016; Zvezdanova et al., 2020).

### 2.3 Statistical methods

Statistical analysis was performed using SPSS software (Version 17.0). Categorical variables are described as frequencies and percentages, whereas continuous variables with normal distributions are described as the means and standard deviations. Pearson’s chi-square test or Fisher’s exact test (if the expected values were less than five) was used to compare categorical variables between different groups. Multivariable logistic regression was performed to evaluate the factors independently associated with mortality at the 3, 6, 9, and 12-month follow-up timepoints. The life table method was used to estimate survival rates at the 3, 6, 9, and 12-month follow-up timepoints. Statistical significance was set at p < 0.05.

## 3 Results

### 3.1 Demographic and clinical characteristics of the patients

A total of 76 patients, 70 males and six females, were included in this study. The median age of the patients was 37 years (interquartile range, 31-48 years). Of the 76 patients, 64 were northerners and 12 were southerners (The northern and southern parts of China are divided by the Qinling Mountains and the Huaihe River. People from the southern part of the Qinling Mountains are called southerners, whereas those from the north are called northerners). Forty-one patients lived urban areas and 35 were from rural areas. The demographic and clinical characteristics of the study population are summarized in **Table 1** according to their survival status at 3, 6, 9, and 12 months. Regarding the clinical characteristics of the survival and death groups at each follow-up timepoint, the initiation time of ART in the death group was later than that in the survival group, indicating that the later the initiation of ART after HIV infection, the higher the probability of death. The rates of oral candidiasis, bacterial pneumonia, cytomegalovirus, and toxoplasma infections at 3 months were higher in the death group than in the survival group. At the 12-month timepoint, the proportion of rural residents in the death group was higher than that in the survival group. Patients with an occupation associated with a high educational level had a lower mortality rate than those with low-skill occupations. The proportions of farmers and unemployed people in the death group were higher than those in the survival group.

**Table 1:** Demographic and clinical characteristics of the patients.

	n (%)	Three months			Six months			Nine months			Twelve months		
		Death (31,40.8%)	Survival (45,59.2%)	P	Death (37, 48.7%)	Survival (39, 51.3%)	P	Death (38,50.0%)	Survival (38,50.0%)	P	Death (41,53.9%)	Survival (35,46.1%)	P
**Mean age(years)**		39.8 ± 9.7	38.1 ± 12.5	0.114	39.4 ± 10.9	38.2 ± 12.0	0.288	39.6 ± 10.8	38.0 ± 12.0	0.294	40.3 ± 11.2	37.9 ± 11.5	0.773
**Sex**				0.964			1.000			1.000			1.000
Male	70 (92.1%)	28 (90.3%)	42 (93.3%)		34 (91.9%)	36 (92.3%)		35 (92.1%)	35 (92.1%)		38 (92.7%)	32 (91.4%)	
Female	6 (7.9%)	3 (9.7%)	3 (6.7%)		3 (8.1%)	3 (7.7%)		3 (7.9%)	3 (7.9%)		3 (7.3%)	3 (8.6%)	
**District of origin**				0.801			0.921			0.529			0.740
South	12 (15.8%)	4(12.9%)	8 (17.8%)		6 (16.2%)	6 (15.4%)		7 (18.4%)	5 (13.2%)		7 (17.1%)	5 (14.3%)	
North	64 (84.2%)	27(87.1%)	37 (82.2%)		31 (83.8%)	33 (84.6%)		31 (81.6%)	33 (86.8%)		34 (82.9%)	30 (85.7%)	
**Residence**				0.202			0.173			0.107			0.018*
Urban	41 (53.9%)	14 (45.2%)	27 (60.0%)		17 (45.9%)	24 (61.5%)		17 (44.7%)	24 (63.2%)		17 (41.5%)	24 (68.6%)	
Rural	35 (46.1%)	17 (54.8%)	18 (40.0%)		20 (50.1%)	15 (38.5%)		21 (55.3%)	14 (36.8%)		24 (58.5%)	11 (31.4%)	
**Occupation**				0.304			0.153			0.078			0.006*
Serving staff	27 (35.5%)	8 (25.8%)	19 (42.2%)		10 (27.0%)	17 (43.6%)		10 (26.4%)	17 (44.7%)		10 (24.4%)	17 (48.5%)	
Retired personnel	4 (5.3%)	1 (3.2%)	3 (6.7%)		1 (2.7%)	3 (7.7%)		1 (2.6%)	3 (7.9%)		1 (2.4%)	3 (8.6%)	
Self-employed	3 (3.9%)	0	3 (6.7%)		0	3 (7.7%)		0	3 (7.9%)		0	3 (8.6%)	
Freelancer	6 (8.0%)	4 (12.9)	2 (4.4%)		4 (10.9%)	2 (5.1%)		4 (10.5%)	2 (5.3%)		4 (9.8%)	2 (5.7%)	
Unemployed	15 (19.7%)	7 (22.6%)	8 (17.8%)		9 (24.3%)	6 (15.4%)		9 (23.7%)	6 (15.7%)		10 (24.4%)	5 (14.3%)	
Peasant	18 (23.7%)	10 (32.3%)	8 (17.8%)		12 (32.4%)	6 (15.4%)		13 (34.2%)	5 (13.2%)		15 (36.6%)	3 (8.6%)	
Student	3 (3.9%)	1 (3.2%)	2 (4.4%)		1 (2.7%)	2 (5.1%)		1 (2.6%)	2 (5.3%)		1 (2.4%)	2 (5.7%)	
**Clinical manifestations**				0.510			0.439			0.439			0.663
Fever	43 (56.6%)	15 (48.4%)	28 (62.2%)		18 (48.7%)	25 (64.1%)		18 (47.4%)	25 (65.8%)		21 (51.2%)	22 (62.9%)	
Headache	38 (50.0%)	18 (58.1%)	20 (44.4%)		21 (56.8%)	17 (43.6%)		21 (55.3%)	17 (44.7%)		22 (53.7%)	16 (45.7%)	
Vomiting	14 (18.4%)	4 (12.9%)	10 (22.2%)		4 (10.8%)	10 (25.6%)		4 (10.5%)	10 (26.3%)		5 (12.2%)	9 (25.7%)	
Cough	12 (15.8%)	5 (16.1%)	7 (15.6%)		5 (13.5%)	7 (18.0%)		5 (13.2%)	7 (18.4%)		7 (17.1%)	5 (14.3%)	
Diarrhea	4 (5.3%)	3 (9.7%)	1 (2.2%)		3 (8.1%)	1 (2.6%)		3 (7.9%)	1 (2.6%)		3 (7.3%)	1 (2.9%)	
Impaired vision	3 (4.0%)	1 (3.2%)	2 (4.4%)		1 (2.7%)	2 (5.1%)		1 (2.6%)	2 (5.3%)		1 (2.4%)	2 (5.7%)	
Hearing loss	1 (1.3%)	1 (3.2%)	0		1 (2.7%)	0		1 (2.6%)	0		1 (2.4%)	0	
**Route of infection**				0.339			0.527			0.492			0.484
Homosexual	16 (21.0%)	4 (12.9%)	12 (26.7%)		6 (16.2%)	10 (25.6%)		6 (15.8%)	10 (26.3%)		7 (17.1%)	9 (25.7%)	
Heterosexual	12 (15.8%)	5 (16.1%)	7 (15.5%)		7 (18.9%)	5 (12.9%)		7 (18.4%)	5 (13.2%)		8 (19.5%)	4 (11.4%)	
Unknown	48 (63.2%)	22 (71.0%)	26 (57.8%)		24 (64.9%)	24 (61.5%)		25 (65.8%)	23 (60.5%)		26 (63.4%)	22 (62.9%)	
**Duration of HIV infection**				0.072			0.257			0.239			0.452
<=3 months	54 (71.1%)	18 (58.1%)	36 (80.0%)		23 (62.2%)	31 (79.5%)		23 (60.5%)	31 (81.6%)		26 (63.4%)	28 (80.0%)	
4-12 months	8 (10.5%)	3 (9.7%)	5 (11.1%)		4 (10.8%)	4 (10.2%)		5 (13.2%)	3 (7.9%)		5 (12.2%)	3 (8.6%)	
1-3 years	5 (6.6%)	4 (12.9%)	1 (2.2%)		4 (10.8%)	1 (2.6%)		4 (10.5%)	1 (2.6%)		4 (9.8%)	1 (2.8%)	
4-9 years	9 (11.8%)	6 (19.3%)	3 (6.7%)		6 (16.2%)	3 (7.7%)		6 (15.8%)	3 (7.9%)		6 (14.6%)	3 (8.6%)	
Laboratory examination													
CD4 (cells/μL)				0.614			0.450			0.345			0.389
≤50	60 (79.0%)	26 (83.9%)	34 (75.6%)		31 (83.8%)	29 (74.4%)		32 (84.2%)	28 (73.7%)		34 (82.9%)	26 (74.3%)	
51-200	14 (18.4%)	5 (16.1%)	9 (20.0%)		6 (16.2%)	8 (20.5%)		6 (15.8%)	8 (21.0%)		7 (17.1%)	7 (20.0%)	
201–500	2 (2.6%)	0	2 (4.4%)		0	2 (5.1%)		0	2 (5.3%)		0	2 (5.7%)	
>500	0	0	0		0	0		0	0		0	0	
**Positive culture**				0.321			0.568	0		0.679			0.848
CSF	71 (93.4%)	28 (90.3%)	43 (95.6%)		34 (91.9%)	37 (94.9%)		35 (92.1%)	36 (94.7%)		38 (92.7%)	33 (94.3%)	
Blood	47 (61.8%)	21 (67.7%)	26 (57.8%)		24 (64.9%)	23 (59.0%)		25 (65.8%)	22 (57.9%)		26 (63.4%)	21 (60.0%)	
**Positive cryptococcal antigen test**				0.721			0.563			0.729			0.729
CSF	67 (88.2%)	26 (83.9%)	41 (91.1%)		32 (86.5%)	35 (89.7%)		33 (86.8%)	34 (89.5%)		36 (87.8%)	31 (88.6%)	
Blood	67 (88.2%)	24 (77.4%)	43 (95.6%)		30 (81.1%)	37 (94.9%)		31 (81.6%)	36 (94.7%)		34 (82.9%)	33 (94.3%)	
**Positive Indian ink stain test**	58 (76.3%)	24 (77.4%)	34 (75.6%)		30 (81.1%)	28 (71.8%)		31 (81.6%)	27 (71.1%)		33 (80.5%)	25 (71.4%)	
**(1-3)-β-D glucan pg/ML**				0.749			0.246			0.168			0.467
≤60	36 (47.4%)	14 (45.2%)	22 (48.9%)		15 (40.5%)	21 (53.8%)		15 (39.5%)	21 (58.3%)		21 (51.2%)	15 (42.9%)	
>60	40 (52.6%)	17 (54.8%)	23 (51.1%)		22 (59.5%)	18 (46.2%)		23 (60.5%)	17 (41.7%)		20 (48.8%)	20 (57.1%)	
**Co-infection**				0.002*			0.700			0.620			0.504
Syphilis	15 (19.7%)	5 (16.1%)	10 (22.2%)		7 (18.9%)	8 (20.5%)		7 (18.4%)	8 (21.1%)		8 (19.5%)	7 (20.0%)	
Tuberculosis	17 (22.4%)	5 (16.1%)	12 (26.7%)		7 (18.9%)	10(25.6%)		7 (18.4%)	10 (26.3%)		9 (21.9%)	8 (22.8%)	
Talaromyces marneffei	1 (1.3%)	0	1 (2.2%)		0	1 (2.6%)		0	1 (2.6%)		0	1 (2.9%)	
Bacterial pneumonia	41 (53.9%)	20 (64.5%)	21 (46.7%)		21 (56.8%)	20 (51.3%)		21 (55.3%)	20 (52.6%)		22 (53.6%)	19 (54.3%)	
Oral candidiasis	20 (26.3%)	11 (35.5%)	9 (20.0%)		12 (32.4%)	8 (20.5%)		13 (34.2%)	7 (18.4%)		15 (36.6%)	5 (14.2%)	
CMV	38 (50.0%)	16 (51.6%)	22 (48.9%)		19 (51.4%)	19 (48.7%)		19 (50.0%)	19 (50.0%)		20 (48.8%)	18 (51.4%)	
EBV	48 (63.2%)	16 (51.6%)	32 (71.1%)		21 (56.8%)	27 (69.2%)		22 (57.9%)	26 (68.4%)		24 (58.5%)	24 (68.6%)	
Toxoplasma	3 (3.9%)	3 (9.7%)	0		3 (8.1%)	0		3 (7.9%)	0		3 (7.3%)	0	
Herpes zoster	6 (7.9%)	2 (6.5%)	4 (8.9%)		3 (8.1%)	3 (7.7%)		3 (7.9%)	3 (7.9%)		3 (7.3%)	3 (8.6%)	
**Number of opportunistic infections**				0.049*			0.140			0.168			0.168
one	2 (2.6%)	2 (6.4%)	0		2 (5.4%)	0		2 (5.3%)	0		2 (4.9%)	0	
two	18 (23.7%)	10 (32.3%)	8 (17.8%)		11 (29.7%)	7 (18.0%)		11 (28.9%)	7 (18.4%)		12 (29.3%)	6 (17.1%)	
more than three	56 (73.7%)	19 (61.3%)	37 (82.2%)		24 (64.9%)	32 (82.0%)		25 (65.8%)	31 (81.6%)		27 (65.8%)	29 (82.9%)	
**Timing of ART initiation**				0.002*			0.006*			0.010*			0.010*
Within 6 months	44 (57.9%)	11 (35.5%)	33 (73.4%)		15 (40.6%)	29 (74.4%)		16 (42.1%)	28 (73.8%)		18 (43.9%)	26 (74.3%)	
After 6 months	11 (14.5%)	5 (16.1%)	6 (13.3%)		6 (16.2%)	5 (12.8%)		6 (15.8%)	5 (13.1%)		6 (14.6%)	5 (14.3%)	
Not initiated	21 (27.6%)	15 (48.4%)	6 (13.3%)		16 (43.2%)	5 (12.8%)		16 (42.1%)	5 (13.1%)		17 (41.5%)	4 (11.4%)	
**Treatment of C*ryptococcus***				0.022*			0.090			0.119			0.262
Monotherapy	6 (7.9%)	4 (12.9%)	2 (4.4%)		4 (10.8%)	2 (5.1%)		4 (10.5%)	2 (5.3%)		4 (9.7%)	2 (5.7%)	
Combination therapy	67 (88.2%)	24 (77.4%)	43 (95.6%)		30 (81.1%)	37 (94.9%)		31 (81.6%)	36 (94.7%)		34 (82.9%)	33 (94.3%)	
Uninitiated	3 (3.9%)	3 (9.7%)	0		3 (8.1%)	0		3 (7.9%)	0		3 (7.4%)	0	

Categorical variables are presented as frequencies (%).

Timing of ART initiation: initiation of antiretroviral therapy after HIV infection.

P value for the comparison of the total number of deaths versus the number of survivors at the 3-, 6-, 9-, and 12-month timepoints.

*p < 0.05.

### 3.2 Correlation between the time of ART initiation and the timing of treatment for cryptococcal infections

**Figure 1:**
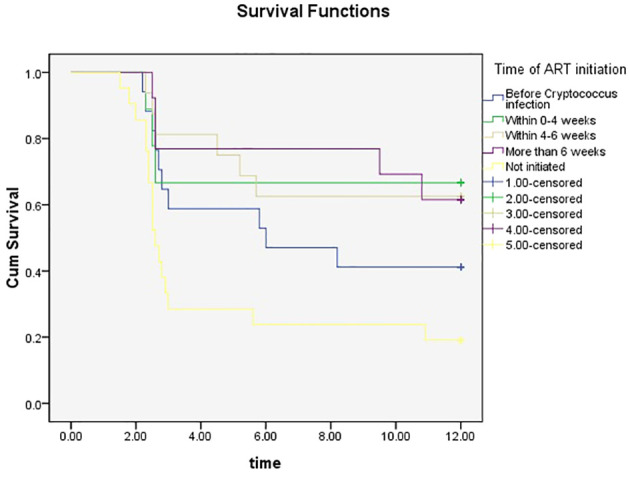
KM survival curve at different ART initiation times.

Analysis of the correlation between the time was ART initiated and the time at which treatment for cryptococcal infection was initiated showed that 17 (22.36%) patients started ART before they got infected with Cryptococcus, 9 (11.84%) started ART within 0-4 weeks of starting treatment for cryptococcal infection, 16 (21.05%) started ART within 4-6 weeks of starting treatment for cryptococcal infection, 13 (17.11%) started ART after more than 6 weeks after the start of treatment for cryptococcal infection, and 21 (27.63%) never started ART. Initiation of ART within 4-6 weeks of starting treatment for Cryptococcus infection was associated with a lower mortality rate at the 3-month follow-up timepoint (P=0.010). Initiation of ART >6 weeks after treatment for Cryptococcus infection was associated with a lower mortality rate at the 6- and 9-month follow-up timepoints (P=0.020, 0.016). Initiation of ART within 0-4 weeks of treatment for Cryptococcus infection was associated with lower mortality rate the 12-month timepoint (P=0.026) (**Table 2**, **Figure 1**).

**Table 2:** Analysis of the correlation between the time of ART initiation and the timing of treatment for cryptococcal infections.

Time of ART initiation		3-month		6-month		9-month		12-month	
		Survival (45,59.2%)	Death (31,40.8%)	Survival (39,51.3%)	Death (37,48.7%)	Survival (38,50.0%)	Death (38,50.0%)	Survival(35,46.1%)	Death(41,53.9%)
Before *Cryptococcus* infection	17 (22.36%)	10 (58.82%)	7 (41.18%)	8 (47.06%)	9 (52.94%)	7 (41.18%)	10 (58.82%)	7 (41.18%)	10 (58.82%)
Within 0-4 weeks	9 (11.84%)	6 (66.67%)	3 (33.33%)	6 (66.67%)	3 (33.33%)	6 (66.67%)	3 (33.33%)	6 (66.67%)	3 (33.33%)
Within 4-6 weeks	16 (21.05%)	13 (81.25%)	3 (18.75%)	10 (62.5%)	6 (37.5%)	10 (62.5%)	6 (37.5%)	10 (62.5%)	6 (37.5%)
More than 6 weeks	13 (17.11%)	10 (76.92%)	3 (23.08%)	10 (76.92%)	3 (23.08%)	10 (76.92%)	3 (23.08%)	8 (61.54%)	5 (38.46%)
Not initiated	21 (27.63%)	6 (28.57%)	15 (71.43%)	5 (23.81%)	16 (76.19%)	5 (23.81%)	16 (76.19%)	4 (19.05%)	17(80.95%)
P		0.010 *		0.020*		0.016*		0.026*	

ART, antiretroviral therapy.

P value for the comparison of the total number of deaths versus the number of survivors at the 3-, 6-, 9-, and 12-month follow-up timepoints. *p < 0.05.

### 3.3 Multivariate regression analysis of prognostic factors for HIV-infection complicated with cryptococcal infection

Multivariate regression analysis showed that time of ART initiation was an independent risk factor for HIV-infection complicated with cryptococcal infection at the four follow-up timepoints. Specifically, the results showed that the later the time of ART initiation, the worse the prognosis. The duration of HIV infection was a risk factor for poor prognosis at the 3-month timepoint. Occupation was the risk factor for poor prognosis at the 12-month follow-up timepoint, the lower the educational level required for the occupation, the higher the risk of death. The results are shown in **Tables 3**-**5**.

**Table 3:** Multivariable logistic regression analysis of the risk factors for 3-month mortality in patients with HIV infection complicated with cryptococcal infection.

	3-month		Multivariate analysis	
	Death (31,40.79%)	Survival (45,59.21%)	P	OR (95%CI)
**Duration of HIV infection**			0.072	
<=3 months	18 (58.06%)	36 (80.00%)		
4-12 months	3 (9.67%)	5 (11.11%)	0.085	6.325 (0.776-51.585)
1-3 years	4 (12.9%)	1 (2.22%)	0.033	68.511 (1.392-3373.140)
4-9 years	6 (19.35%)	3 (6.67%)	0.020	29.63 (1.689-519.846)
**Time of ART initiation**			0.010	
Within 6 months	11 (35.48%)	33 (73.33%)		
After 6 months	5 (16.13%)	6 (13.33%)	0.265	0.208 (0.013-3.219)
Not initiated	15 (48.39%)	6 (13.33%)	0.000	30.516 (5.012-185.779)

Time of ART initiation= initiation of antiretroviral therapy after HIV infection.

**Table 4:** Multivariable logistic regression analysis of the risk factors for 6- and 9-month mortality in patients with HIV infection complicated with cryptococcal infection.

	6-month		Multivariate analysis		9-month		Multivariate analysis	
	Death (37,48.68%)	Survival (39,51.32%)	P	OR (95%CI)	Death (38,50.0%)	Survival (38,50.0%)	P	OR (95%CI)
**Time of ART initiation**			0.007				0.015	
Within 6 months	15 (40.54%)	29 (74.36%)			16 (42.11%)	28 (73.68%)		
After 6 months	6 (16.22%)	5 (12.82%)	0.154	2.677 (0690-10.380)	6 (15.78%)	5 (13.16%)	0.277	2.100 (0.552-7.991)
Not initiated	16 (43.24%)	5 (12.82%)	0.002	6.692 (2.005-22.334)	16 (42.11%)	5 (13.16%)	0.004	5.600 (1.726-18.173)

Time of ART initiation = initiation of antiretroviral therapy after HIV infection.

**Table 5:** Multivariable logistic regression analysis of the risk factors for 12-month mortality in patients with HIV infection complicated with cryptococcal infection.

	12-month		Multivariate analysis	
	Death (41,53.95%)	Survival (35,46.05%)	P	OR (95%CI)
**Occupation**			0.076	
Serving staff	10 (24.39%)	17 (48.57%)	0.731	1.67 (0.089-31.166)
Retired personnel	1 (2.44%)	3 (8.57%)	0.887	0.761 (0.017-33.216)
Self-employed	0	3 (8.57%)	0.999	0.000
Freelancer	4 (9.76%)	2 (5.71%)	0.462	3.632 (0.117-112.534)
Unemployed	10 (24.39%)	5 (14.29%)	0.271	5.547 (0.262-117.331)
Peasant	15 (36.59%)	3 (8.57%)	0.066	18.377 (0.822-410.655)
Student	1 (2.44%)	2 (5.71%)		
**Time of ART initiation**			0.011	
Within 6 months	18 (43.90%)	26 (74.29%)		
After 6 months	6 (14.63%)	5 (14.29%)	0.796	1.225 (0.263-5.699)
Uninitiated	17 (41.46%)	4 (11.43%)	0.003	9.959 (2.182-45.467)

Time of ART initiation = initiation of ntiretroviral therapy after HIV infection.

### 3.4 Analysis of the survival of patients with HIV-infection complicated with cryptococcal infection

**Figure 2:**
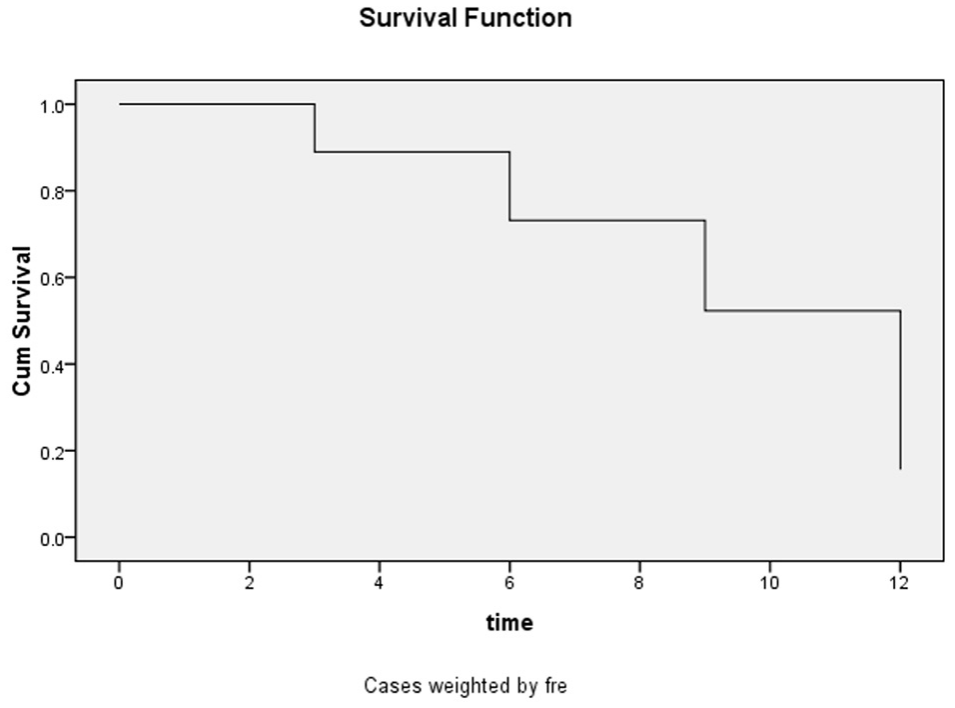
Survival function curves for the entire 12-month follow-up period.

The estimated survival rate of patients with HIV-infection with cryptococcal infection, which was performed using the life table method, was 0.89, 0.73, 0.52, and 0.16 at the 3-, 6-, 9-, and 12- month follow-up timepoints, and the median duration of survival was 9.19 months. The survival curves are shown in **Figure 2**.

## 4 Discussion

*Cryptococcus neoformans* was ranked the first fungus of concern in the critical priority group in the Priority List of Pathogenic Fungi published by the WHO (World Health Organization, 2022). *Cryptococcus neoformans* is highly likely to cause invasive infections in individuals with impaired immune function, especially HIV-infected individuals. *Cryptococcus neoformans* is neurophilic, and cryptococcal meningitis is common after infection. The mortality rate of cryptococcal meningitis in low-and middle-income countries can be as high as 70% (Skipper et al., 2022). The number of people infected with HIV in China has been increasing every year (Nation Health Commission of China, 2023). Although antiretroviral treatment regimens can improve the quality of life of patients with HIV infection, no complete cure for AIDS has been discovered yet. The diagnosis and treatment of HIV infection combined with cryptococcal infection in clinical practice is challenging. Thus, the clinical characteristics and related prognostic factors of HIV infection combined with cryptococcal infection require further investigation to facilitate the improvement of management and patient outcomes. The present study was conducted to advance our understanding of HIV infection complicated with cryptococcal infection and provide valuable information that can optimize its management.

We retrospectively analyzed the case data and relevant laboratory test results of patients with HIV infection complicated with cryptococcal infections between 2013 and 2023. The results showed that most of the patients were middle-aged and young men. The peak age range for HIV infection combined with cryptococcal infection was 31 to 40 years, which is slightly different from the 31-50 years peak age range for HIV infection reported in previous studies (Oliveira et al., 2023). Data analysis showed that the proportion of patients living in urban areas was slightly higher than that of patients living in rural areas. Moreover, patients living in urban areas had better prognoses than those from rural areas. This may be attributed to the fact that patients living in urban areas have access to better medical resources, making the diagnosis and treatment of diseases easier. This suggests that the medical environment affects patient prognosis, and that high-quality medical resources are beneficial for disease diagnosis and treatment, which is consistent with the findings of other studies (Klein et al., 2020). Classification of the patients according to the occupational background revealed that patients with higher education levels and stable jobs had lower mortality rates than those with lower education levels and no stable job. Education level affects a patient’s understanding and prognosis of the disease. Regarding the rout of infection, most patients in our study cohort did not indicate the source of infection, and those who provided the information reported that homosexual behavior was the main route of HIV infection. HIV-positive individuals often conceal their identities, which makes them susceptible to being invisible disease carriers. Multivariate regression analysis showed that the duration of HIV infection was an independent risk factor for the 3-month prognosis of HIV infection complicated with cryptococcal infection. The results also showed that the shorter the duration of HIV infection, the better the prognosis. China is a populous country among developing countries, and the distribution of medical resources is still uneven. Most of the populace are still low-income earners, and the overall perception of HIV infection still needs to be improved. Based on the current status and epidemic trends of HIV infection, it is important to educate the general public on prevention of HIV infection. Improving public awareness of HIV infection can promote its prevention and treatment.

In the present study, approximately 70% of the patients diagnosed with HIV infection were infected with *Cryptococcus* within 3 months. Of these, 79% had a CD4 cell count lower than 50 cells/µL, indicating that most of the patients may have had latent *Cryptococcus* colonization; thus, *Cryptococcus* was activated once the immune system was weakened (Lazera et al., 2000). In addition, 95% (74/76) of the patients were diagnosed with cryptococcal meningitis, with fever and headache as the first symptoms. Regarding laboratory test results, 93%, 88%, and 24% of the patients had positive *Cryptococcus* culture in CSF, blood, and both CSF and blood samples, respectively. This finding supports the neurotropism of *Cryptococcus*, which easily breaks through the blood–brain barrier and causes brain infection after entering the blood. Regarding methods used for the detection of *Cryptococcus*, culture and cryptococcal antigen (CrAg) detection has a high positive rate and good consistency rate, whereas the traditional India ink staining method has a relatively low positive rate owing to the method being personnel-dependent. The CrAg test is simple, rapid, and highly accurate for screening cryptococcal infections and is very helpful for timely clinical diagnosis.

The mortality rate of HIV-related cryptococcal meningitis higher than 15%, even with access to sufficient medical resources (Skipper et al., 2019; Rajasingham et al., 2022; Boulware and Jarvis, 2023). Early initiation of ART can quickly restore immune function in immunosuppressed patients, eliminate *Cryptococcus*, and prevent other fatal opportunistic infections. However, if ART is initiated while a patient has a cryptococcal infection, it is necessary to balance its benefits with the risk of immune reconstitution inflammatory syndrome (IRIS) (Boulware et al., 2010). The findings of the final COAT trial led by Boulware, which were included in the guidelines for Diagnosing, Preventing and Managing Cryptococcal Disease Among Adults, Adolescents and Children Living with HIV approved by WHO, showed that early initiation of ART is associated with an absolute mortality rate 15% higher than that for delayed initiation of ART (Boulware et al., 2014); however, this trial was conducted in Africa, and the patient population is different from those in high-income regions. To gain a deeper understanding of the impact of timing of ART, Ingle led a study based on three US/European cohorts and found that early initiation of ART did not significantly increase mortality in high-income areas (Boulware and Jarvis, 2023). These findings indicate that the optimal time for initiation of ART for cryptococcal meningitis in HIV-infected individuals remains controversial. In addition, whether further clinical trials are needed and whether the guidelines should be changed and refined are still debatable. In the present study, cryptococcal infection was used as the observation point for timing of ART initiation, and the results showed that patients who initiated ART within 4-6 weeks of starting treatment for cryptococcal infection had a lower mortality rate at 3 months than those who initiated ART at other timepoints. This is slightly different from the general guideline that recommends initiation of ART after 4-6 weeks of antifungal therapy for cryptococcal coinfection; notably, this recommendation does not include a specific time interval. Patients who initiated ART more than 6 weeks after the start of treatment for cryptococcal infection showed lower mortality rates at 6 and 9 months than those who initiated ART at other timepoints. Patients who initiated ART within 4 weeks of starting cryptococcal therapy showed lower mortality at 12 months than those who initiated ART at other timepoints. Our study provides reference for the selection of ART therapy timing for HIV/AIDS patients with cryptococcal infection, and provides a basis for the revision of treatment guidelines for HIV/AIDS patients with cryptococcal infection.

In addition, when HIV infection was used as the observation point for initiation of ART, time of ART initiation was found to be an independent risk factor for *Cryptococcus* infection in HIV-infected patients at 3, 6, 9, and 12 months. The later the initiation of ART after HIV infection, the worse the prognosis. Although it is necessary to balance the benefits of ART in patients with HIV and cryptococcal infections with the risks of IRIS, the results of the present study suggest that HIV-infected individuals should not start ART too late, even if they have cryptococcal infection. Starting ART within 6 months of HIV infection is associated with a better prognosis than starting it later or not starting it at all, implying that the earlier ART is administered after HIV infection, the better the overall survival. The present study provides data regarding the timing of ART treatment in real-world settings and a reference for the determination of ART timing guidelines. However, further research is necessary to determine the optimal timing of ART initiation. The limitation of this study include it is an observational design, insufficient sample size, not long enough follow-up time, potential biases and limited data. In the future, the study cohort can be further expanded by increasing observation cases, monitoring indicators, and follow-up time to obtain more comprehensive and in-depth studies.

## 5 Conclusions

HIV-infection complicated with cryptococcal infection is a critical case with a median survival time of less than 1 year. This study demonstrated that ART is a key factor that affects the prognosis of such cases. Specifically, the results indicated that the later ART after HIV infection is started, the worse the prognosis of cryptococcal infection in HIV-infected patients. The time of ART initiation and the timing of treatment for cryptococcal infections should be further refined and balanced based on different clinical courses. Clinicians should pay closer attention to cryptococcal infection in patients with HIV infection and actively initiate ART and other appropriate measures based on each patient’s clinical condition.

## Data Availability

All relevant data is contained within the article: The original contributions presented in the study are included in the article material, further inquiries can be directed to the corresponding author/s.
